# Epigenetic Alterations in Human Papillomavirus-Associated Cancers

**DOI:** 10.3390/v9090248

**Published:** 2017-09-01

**Authors:** David Soto, Christine Song, Margaret E. McLaughlin-Drubin

**Affiliations:** Division of Infectious Disease, Department of Medicine, Brigham & Women’s Hospital, Harvard Medical School, 181 Longwood Avenue, Boston, MA 02115, USA; dsoto7@bwh.harvard.edu (D.S.); cksong@partners.org (C.S.)

**Keywords:** Human papillomavirus, HPV, cervical cancer, epigenetics, histone

## Abstract

Approximately 15–20% of human cancers are caused by viruses, including human papillomaviruses (HPVs). Viruses are obligatory intracellular parasites and encode proteins that reprogram the regulatory networks governing host cellular signaling pathways that control recognition by the immune system, proliferation, differentiation, genomic integrity, and cell death. Given that key proteins in these regulatory networks are also subject to mutation in non-virally associated diseases and cancers, the study of oncogenic viruses has also been instrumental to the discovery and analysis of many fundamental cellular processes, including messenger RNA (mRNA) splicing, transcriptional enhancers, oncogenes and tumor suppressors, signal transduction, immune regulation, and cell cycle control. More recently, tumor viruses, in particular HPV, have proven themselves invaluable in the study of the cancer epigenome. Epigenetic silencing or de-silencing of genes can have cellular consequences that are akin to genetic mutations, i.e., the loss and gain of expression of genes that are not usually expressed in a certain cell type and/or genes that have tumor suppressive or oncogenic activities, respectively. Unlike genetic mutations, the reversible nature of epigenetic modifications affords an opportunity of epigenetic therapy for cancer. This review summarizes the current knowledge on epigenetic regulation in HPV-infected cells with a focus on those elements with relevance to carcinogenesis.

## 1. Introduction

Approximately 15–20% of the 12.7 million incident cancer cases per year have a viral etiology [1,2]. Carcinogenesis is a complex, multi-step process, and oncogenic viruses, including high-risk human papillomaviruses (HPVs), the Epstein-Barr virus (EBV), the hepatitis B virus (HBV), the hepatitis C virus (HCV), human T cell lymphotrophic virus-1 (HTLV-1), Kaposi’s sarcoma herpesvirus (KSHV), and the Merkel cell polyoma virus (MCV), contribute to different steps of this process (reviewed in [3]). Viruses are obligatory intracellular parasites and encode proteins that reprogram the regulatory networks governing host cellular signaling pathways that control recognition by the immune system, proliferation, differentiation, genomic integrity, and cell death. The study of oncogenic viruses, as well as the manner in which they target regulatory nodes, has been key to the understanding of the etiology of several human cancers. It has led to the development of prophylactic vaccines for HBV along with the most abundant low- and high-risk HPVs. Given that key proteins in these regulatory networks are also subject to mutation in non-virally associated diseases and cancers, the study of oncogenic viruses has also been instrumental to the discovery and analysis of many fundamental cellular processes, including mRNA splicing, transcriptional enhancers, oncogenes and tumor suppressors, signal transduction, immune regulation, and cell cycle control (reviewed in [3,4]). More recently, tumor viruses, in particular HPV, have proven themselves invaluable in the study of the cancer epigenome.

The concept that cancer is equally an epigenetic and a genetic disease has been increasingly validated during the past decade, particularly since the advent of whole-genome approaches. Epigenetic abnormalities in cancer involve aberrations in virtually every aspect of chromatin biology, including post-translational modifications of histone proteins, DNA methylation, chromatin remodeling, and non-coding RNAs (ncRNAs). The cancer epigenome harbors numerous abnormalities that distinguish it from its normal counterpart (reviewed in [5]). Aberrations in virtually every aspect of chromatin biology have been identified in cancer-harboring epigenetic abnormalities, including post-translational modifications of histone proteins, DNA methylation, chromatin remodeling, and ncRNAs. For example, aberrant methylation patterns and histone modifications are found in both virus-associated and non-viral cancers [6,7,8]. Indeed, viral oncoproteins can induce the expression of, as well as interact with, DNA methyltransferases (DNMTs) and histone-modifying enzymes, including histone deacetylases (HDACs), histone acetyltransferases (HATs), histone methyltransferases (HMTs), and histone demethylases (reviewed in [9,10]). Moreover, viral oncoproteins can also alter the activity of chromatin-remodeling, complex-associated proteins and miRNA processing-associated proteins (reviewed in [9,10]). In addition to causing alterations in the host epigenome, the tumor virus genome itself also undergoes epigenetic modification (reviewed in [11]).

A full understanding of the cancer epigenome has numerous translational implications. It is evident that cancer cells have global epigenome changes involving entire pathways. These epigenetic alterations are often found early in tumorigenesis and are likely to be key initiating events in certain cancers (reviewed in [12]). In addition to tumor initiation, epigenetic events also contribute to tumor progression (reviewed in [5]). Virus-associated cancers present unique experimental systems to determine what role epigenetic modifications play in carcinogenesis. Similar to hematological tumors that are often driven by a single initiating mutation, virus-associated cancers are initiated by a uniform oncogenic hit of viral oncogene expression. This has been quite impressively demonstrated for HPV-associated cervical cancers where cancer initiation and progression are driven by the expression of the E6 and E7 oncogenes (reviewed in [13]). Much has already been learned from detailed molecular analyses of epigenetic mechanisms in virus-induced tumorigenesis (reviewed in [9]). The study of tumor viruses such as HPV should continue to provide answers regarding the importance of epigenetic alterations in viral cancers as well as, hopefully, non-viral-associated cancers. It is predicted that epigenetic factors, including readers, writers, and erasers, that are targeted by HPV, are important drug targets for both viral and non-viral associated cancers. This includes factors that are induced by HPV or triggered in response to viral infection/viral protein expression. The reversibility of epigenetic modifications makes such epigenetic factors ideal therapeutic targets. Several drugs targeting chromatin modifiers are already in use in the clinic (reviewed in [5]).

### Human Papillomaviruses

HPVs are small, double-stranded DNA virus members of the *Papillomaviridae,* a large family with a tropism for squamous epithelium. To date, more than 200 HPV types have been described, which are divided into cutaneous and mucosal HPVs based on the tissue they infect. The mucosal HPVs are clinically classified as “high-risk” and “low-risk” based on the propensity for malignant progression. Low-risk HPVs, such as HPVs 6 and 11, cause benign genital warts, while high-risk HPVs cause intraepithelial lesions that are at risk for malignant progression. Infection with high-risk HPVs are associated with approximately 5% of all human cancers, in particular with cervical carcinoma, the third most common cancer in women worldwide [1,14]. HPV infections are also frequently associated with other anogenital cancers, including anal, vulvar, vaginal, and penile cancers, as well as oropharyngeal cancers [15,16]. While prophylactic vaccination prevents infections with HPV types represented in the vaccine, no therapeutic efficacy is associated with these vaccines. In addition to the fact that HPV-associated cervical cancers arise years after initial infection, vaccination rates are low in many countries. Therefore, it will be decades before the current vaccination efforts have a measurable impact on the incidence of HPV-associated tumors [17].

The viral E6 and E7 proteins are consistently expressed in HPV-associated lesions and cancers, and are the major drivers of cell transformation (reviewed in [4,13]). The HPV E6 and E7 proteins lack enzymatic activities and instead function by associating with host cellular proteins. These proteins reprogram cellular signal transduction pathways (reviewed in [18]), causing alterations in the “hallmarks of cancer” [4,19]. Notably, high-risk mucosal HPV E6 and E7 proteins, respectively, target p53 and retinoblastoma (pRB) tumor suppressors; these tumor suppressor pathways are also rendered dysfunctional by mutation in almost all human solid tumors [20,21]. High-risk HPV E6 and E7 also interact with a number of other proteins, such as transcription factors, thus altering cellular gene expression. In addition to targeting specific transcriptional programs, the HPV E6 and E7 oncoproteins can globally alter the transcriptional competence of the infected cells by affecting epigenetic control mechanisms. Indeed, epigenetic alterations such as changes in the DNA methylation pattern of the viral and host genomes, as well as changes in histone modifications, are often found associated with HPV infection and cervical carcinogenesis. This article focuses on HPV-induced changes in these epigenetic control mechanisms, including DNA methylation, histone modifications, chromatin remodeling proteins, and ncRNAs.

## 2. DNA Methylation

DNA methyltransferases (DNMTs) methylate the carbon-5 position of cytosine nucleotides; this covalent modification occurs predominantly on cytosines preceding guanine nucleotides (CpG dinucleotides). In normal cells, methylation of DNA is involved in the regulation of gene expression, including the organization of active and inactive chromatin, tissue-specific gene expression, and genomic imprinting (reviewed in [22]). In contrast, global DNA hypomethylation in repetitive regions and hypermethylation in CpG islands of tumor suppressor gene promoters are frequently observed in tumors [23,24], and the activity of DNMT1, which is the maintenance methyltransferase, is often increased (reviewed in [6,7]). These alterations are also observed in HPV-induced carcinogenesis. HPV E7 binds to DNMT1 and stimulates its DNA methyltransferase activity [25], and may be able to activate transcription of DNMT1 through the pRB/E2F pathway [26], while HPV E6 upregulates DNMT1 by suppression of p53 [27]. As a consequence of the association of HPV E7 with DNMT1, E-cadherin expression is suppressed and adhesion between squamous epithelial cells is reduced [28,29]. Similarly, increased expression of DNMT3A and 3B has also been observed in HPV-positive cells [28,30,31]. The effects of HPV on the DNA methylation machinery have the ability to alter both the host and the viral genome.

### 2.1. HPV Genome Methylation

While methylation of CpG islands in human gene promotors generally represses gene transcription, the methylation of viral DNA both negatively and positively regulates viral gene transcription. Although it is unclear if viral DNA methylation provides a growth advantage to the infected cell, it has been suggested that viral DNA methylation is due to a host defense response to silence viral replication and transcription [32,33,34,35]. HPV gene methylation, particularly in the L1 and L2 genes, varies during the viral life cycle as well as with the disease stage [36,37,38,39,40,41]. Methylation of the upstream regulatory region (URR) appears to be associated with latent infection [42], although results from different studies are inconsistent, possibly due to the integrated or episomal state of the viral genome and/or the stage of the lesion examined. When comparing URR methylation in cervical intraepithelial neoplasia (CIN) and cancer samples compared to normal samples, some studies described decreased methylation [43,44,45], while others showed an increase in URR methylation [33,34,35,41,46]. URR methylation also differs based on type 1 versus type 2 HPV integration [36,37,45,46,47,48]. These differences highlight the need to take into account not only the methodology used to analyze methylation but also the HPV genome and disease status when comparing across studies.

Methylation of the E2 binding sites (E2BSs) in the URR reduces E2 binding, thus deregulating E6 and E7 expression [49], and methylation of E2BSs in reporter plasmids inhibits the transcriptional transactivation activity of E2 in transfected cells [36]. E2 also functions in the initiation of viral DNA replication and in partitioning the viral DNA to the daughter cells during cell division; both of these activities also rely on its ability to bind E2BSs and are thus thought to be affected by the methylation status of the E2BSs. E2BSs in the immortalized HPV16-positive W12 cells are hypomethylated upon differentiation in vivo, providing evidence that the methylation of the E2BSs varies during epithelial differentiation and thus during the viral life cycle [36,42,47]. An analysis of the methylation status of the HPV16 URR in distinct stages of the viral life cycle from patient-derived tissues confirmed a decrease in the methylation of the transcriptional enhancer region of the URR, but also indicated hypermethylation of the E2BSs [42]. Additional studies indicate that methylation of HPV DNA may differentiate between an acute HPV infection and CIN2^+^ (reviewed in [40]). Indeed, it has been proposed that CpG methylation status is a potential biomarker for cervical cancer [50].

### 2.2. Cellular Gene Methylation

Aberrant methylation occurs frequently in cervical cancer, leading to inappropriate gene expression, the activation of oncogenes and transposable elements, loss of imprinting, and the inactivation of tumor suppressor genes (reviewed in [51]). Of note, a number of tumor suppressor genes are hypermethylated in HPV-associated lesions and carcinomas, including *CCNA1* and *hTERT* [8,52,53,54,55,56,57,58,59]. The most frequently methylated genes in cervical cancer are cell adhesion molecule 1 (*CADM1*), cadherin 1 (*CDH1*), death-associated protein kinase 1 (*DAPK1*), *EPB41L3*, *FAM1A4*, myelin and lymphocyte (*MAL*), paired box 1 (*PAX1*), PR domain containing 14 (*PRDM14*,) and telomerase reverse transcriptase (*hTERT*) [52,58,59], however a single gene target has not proven amenable as a biomarker [52] indicating that a panel of methylated genes may be more useful.

## 3. Regulation of Histone Modifications

In addition to DNA methylation, the epigenetic regulation of gene expression is also impacted by histone modifications and the remodeling of nucleosomes. Post-translational modifications of histone tails, including acetylation, methylation, phosphorylation, sumoylation, and ubiquitination, impact the physical state and the transcriptional competence of chromatin. These modifications play a crucial role in the regulation of cellular processes such as stem cell maintenance, cell fate determination and maintenance, cell cycle control, and epigenetic heritability of transcriptional programs (reviewed in [60,61]). Distinct posttranslational modifications on histones, or combinations thereof, characterize transcriptionally active and silent chromatin. In general, transcriptionally active genes are characterized by promoters with unmethylated CpG dinucleotides and nucleosomes. These active genes are arranged such that transcription and regulatory factors are allowed access. Transcriptionally active genes usually have extensive H3 and H4 acetylation and are marked by trimethylation of lysine 4 on histone H3 (H3K4me3), trimethylation of lysine 79 on histone H3 (H3K79me3), ubiquitylation of H2B (H2Bub), and trimethylation of lysine 36 on histone H3 (H3K36me3), while transcriptionally inactive genes are characterized by low levels of acetylation and high levels of trimethylation of lysine 9 on histone H3 (H3K9me3), trimethylation of lysine 27 on histone H3 (H3K27me3), trimethylation of lysine 20 on histone H4 (H4K20me3), and ubiquitylation of lysine 119 on histone H2A (H2AK199ub) (reviewed in [62]). The different patterns of histone modifications associated with distinct transcriptional states are established via interplay between histone readers, writers, and erasers. Enzymes that modify histones and other chromatin components are designated writer proteins, and include HATs, histone methyltransferases (KMTs), and histone ubiquitin ligases; these modifications are reversible and are removed by erasers such as HDACs, histone demethylases, and histone deubiquitinases. The modifications are recognized by reader proteins, which bind to the modified histones and recruit additional proteins [62,63], and ultimately realize the functional translation of the epigenetic mark.

### 3.1. Histone Modification of the Human Papillomavirus Genome

Human papillomavirus genomes are bound by nucleosomes around the viral promoters [64,65,66]. Chromatin immunoprecipitation (ChIP) analysis of the histones bound to the HPV genome throughout the differentiation-dependent viral life cycle demonstrated the presence of acetylated H3 and H4 histones and the dimethylation of lysine 4 on histone H3 (H3K4me2) at the HPV early and late promoters, indicating that they are in an active conformation throughout the viral life cycle [67]. The levels of acetylation and the demethylation of the histones at the early and late promoter regions increase upon differentiation, and the binding of a number of transcription factors was increased upon differentiation [67]. In summary, this study indicated that both the early and late HPV promoter regions are in an active chromatin state throughout the viral life cycle. In a study on the HPV18-positive HeLa cervical cancer cell line, localized distinctions in the status of histone modifications of the chromatin on the HPV18 genome were observed; these correlated with the occupancy of the host transcriptional machinery [41]. The viral E6 and E7 oncoproteins modulate the host epigenetic machinery and histone modification enzymes, which has implications for the epigenetic regulation of both the viral and host genomes, and has implications in both the viral life cycle and the carcinogenic process.

### 3.2. Acetylation

**Figure 1:**
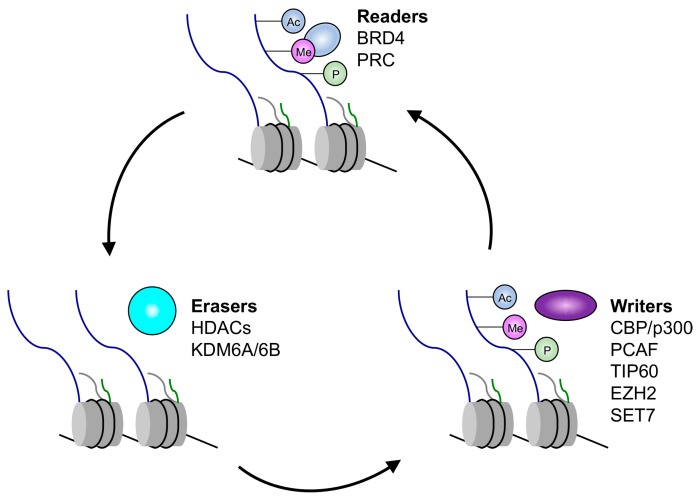
Summary of histone readers, writers, and erasers targeted by human papillomavirus (HPV). Abbreviations: BRD4: bromodomain-containing protein 4; PRC: polycomb repressive complex; CBP/p300: CREB-binding protein/p300; pCAF: p300/CBP-Associated Factor; TIP60: Tat interactive protein, 60 kDa; EZH2: enhancer of zeste homolog 2; SET7: SET domain containing lysine methyltransferase 7; HDACs: histone deacetylases; KDM6A/6B: histone lysine demethylases 6A/6B.

One mechanism by which the HPV E6 and E7 oncoproteins alter the transcriptional competence of infected cells is by associating with and/or modulating the expression, as well as the activities, of histone-modifying and chromatin-remodeling enzymes (Figure 1) [68,69,70,71,72,73,74,75,76,77]. For example, acetylation of lysine residues of histones 3 and 4 (H3 and H4) by HATs leads to transcriptionally active chromatin, while the removal of these marks by HDACs results in transcriptionally repressed chromatin. HPV E6 and E7 can associate with and modulate the activity of the HATs p300 and CBP [70,71,73,78,79,80]; p300/CBP regulates a number of genes [73,81,82,83,84,85,86,87]. HPV E6 inhibits p300/CBP-mediated acetylation of p53 [88], while HPV E7 forms a complex with p300/CBP and pRB, acetylating pRB and decreasing p300/CBP levels [80]. HPV E7 also associates with p300/CBP-Associated Factor (pCAF), reducing its ability to acetylate histones [70] and the steroid-receptor coactivator (SRC1), and abrogating SRC1-associated HAT activity [72]. Moreover, the HPV E7 oncoprotein interacts with class I HDACs [68,69], which function as transcriptional co-repressors by inducing chromatin remodeling via the reversal of acetyl modifications on histone lysine residues. The association of E7 and HDAC1/2 occurs in an RB-independent manner through the intermediary Mi2β, a member of the nucleosome remodeling and histone deactylation (NuRD) complex; the NuRD complex remodels chromatin structure through the deactylation of histones and ATP-dependent nucleosome repositioning [89,90]. The association of E7 and HDAC1/2 does not result in the inhibition of HDAC activity [68], but does play a role in HPV E7-associated transcriptional regulation. For example, this association results in increased levels of E2F2-mediated transcription in differentiating cells, which may affect S-phase progression [91]. Furthermore, HPV E7 can interact with interferon response factor 1 (IRF1) and recruit HDACs to suppress IRF1 transcriptional activity [92,93].

### 3.3. Polycomb Group Proteins and Histone Lysine Modifications

Global levels of the polycomb-regulated H3K27me3 repressive mark are dramatically decreased in HPV16 E7-expressing primary human foreskin keratinocytes and in HPV16-positive cervical lesions and cancers [75,77]. The function of the H3K27me3 mark is exerted by the formation of two polycomb repressive complex (PRC) species, PRC1 and PRC2. PRC2 contains the histone methyltransferase (KMT) EZH2 (KMT6), which places the H3K27me3 mark. The H3K27me3 marked chromatin is occupied by PRC1, and the chromatin is further silenced by mono-ubiquitination of lysine 119 on histone H2A (H2AK119Ub). Gene expression can also be silenced by certain PRC1 complexes in the absence of H3K27me3, as H2AK119Ub is a binding site for L3MBTL2, which establishes repressive structures [94] that play an important role in pluripotent stem cells [95].

PcG proteins regulate both epithelial cell differentiation and the expansion of basal cell pools during the wound healing process [96,97,98], two processes that HPVs may target during the viral life cycle. Thus, it is not surprising that HPVs target components of the PRC machinery (reviewed in [99]). Indeed, HPV16 E7 associates with, as well as potentially modifies, activities of E2F6-containing PRCs and causes a reduction in the number of nuclear E2F6-containing polycomb bodies [76]. Moreover, PcG proteins are likely best known for their role in maintaining stable transcriptional repression of Homeobox (*HOX*) genes during development [100,101], and HOX family members are frequently dysregulated during carcinogenesis, including cervical carcinogenesis and in HPV16 E7-expressing cells [75,102,103,104,105].

While the decrease in H3K27me3 observed in HPV16 E7-expressing cells offered a potential explanation for the decrease in polycomb body number and the dysregulation of *HOX* genes, this decrease is observed despite the fact that the enhancer of the zeste homolog 2 (EZH2) component of the polycomb repressive complex 2 (PRC2) is highly overexpressed in cervical lesions and tumors in an E2F-dependent manner [106]. A number of possible mechanisms have been proposed to explain the seemingly paradoxical finding of decreased H3K27me3 in the presence of increased EZH2. AKT-mediated phosphorylation of EZH2 negatively regulates the enzymatic activity of EZH2 [107], and both HPV16 E6 and E7 activate AKT [108,109]. Thus, it is possible that PRC2-associated EZH2 enzymatic activity is low despite high EZH2 levels in HPV-expressing cells. EZH2 overexpression has also been shown to enhance PRC4 formation [110]. PRC4 causes histone H1K26 deacetylation and methylation [110], which then serves as a binding site for L3MBTL1. Hence, increased EZH2 expression in E7-expressing cells may be predicted to cause enhanced H1K26 methylation. Additionally, another mechanistic explanation for the decrease in H3K27me3 was provided by the finding that the histone lysine demethylases (KDMs) KDM6A (UTX) and KDM6B (JMJD3) are expressed at markedly higher levels in these cells [74,75,77]. Interestingly, cervical cancer cells are dependent on the expression of KDM6A and KDM6B [74,75]. Although KDM6A and KDM6B appear identical with regards to catalytic activities and histone substrate specificities, they have a number of unique biological targets. KDM6B, but not KDM6A, regulate RAS/RAF and HPV E7-induced oncogene-induced senescence (OIS) [74,111,112]. OIS is a cell-intrinsic tumor-suppressive mechanism that protects cells from unrestrained proliferation following an oncogenic insult (reviewed in [113]). In order for a lesion to progress, OIS must be evaded or bypassed, as evidenced by the fact the OIS is observed in premalignant lesions much more than in frank lesions [114]. OIS is signaled through transcriptional upregulation of the p16^INK4A^ tumor suppressor [115]. The p16^INK4A^ tumor suppressor is a biomarker for high-risk, HPV-associated lesions and cancers, and is induced by HPV E7 [116,117]. These high levels of p16INK4A expression are a readout of HPV E7-induced OIS [74]. Interestingly, HPV E7-expressing and some cervical cancer cells are “addicted” to the expression of p16^INK4A^, suggesting that the biological activity of p16^INK4A^ in HPV-associated cancers is more like that of an oncogene, as opposed to its well-established role as a tumor suppressor in most other human cancer types [74].

### 3.4. Histone Arginine Modifications

Histone methylation also takes place on arginine residues, and HPV modulates the activity of two coactivator histone arginine methyltransferases, CARM1 and PRMT1 [118]. HPV E6 downregulates their expression, and these HMTs are needed for HPV E6 to attenuate p53 transactivation [118]. E6 hinders CARM1- and PRMT1-mediated histone methylation at p53-responsive promoters and suppresses p53 binding to DNA [118]. E6 also inhibits SET7, which, in addition to catalyzing H3K4 monomethylation, methylates non-histone proteins, including p53 [119]. HPV E6 downregulates p53K372 mono-methylation, thereby reducing p53 stability [118]. Together, modulation of CARM1, PRMT1, and SET7 provides another mechanism by which HPV alters p53 function.

### 3.5. Epigenetic Readers

Bromodomain-containing protein 4 (Brd4) is a member of the bromodomain and extra-terminal domain (BET) family of chromatin-binding proteins [120] and plays a crucial role in transcription. The bromodomains of Brd4 interact with methylated histones H3 and H4 [121] and mark genes that are expressed shortly after exit from mitosis [122,123]. Brd4 recruits transcription initiation and elongation factors to these genes [124], including the transcriptional elongation factor, p-TEFb [125,126]. Brd4 plays a key role in the transcriptional regulation and replication of papillomaviruses (reviewed in [127]).

The papillomavirus E2 protein interacts with Brd4, stabilizing its association with chromatin [128,129,130,131,132,133]. E2 interacts with the C-terminal domain (CTD) of Brd4, blocking the formation of Brd4-pTEFb [134], and thus acting as an E2-dependent transcriptional repressor of E6 and E7. Brd4 also represses the HPV early promoter, and the binding of Brd4 to the HPV early promoter is dependent on histone H4 acetylation by TIP60 [135]. HPV E6 destabilizes TIP60 in a proteasome-dependent manner, derepressing the early promoter, resulting in HPV oncoprotein expression [135,136].

## 4. Non-Coding RNAs

It has recently become evident that the non-coding portion of the human genome plays an important role in the regulation of the expression of activities of cellular proteins. ncRNAs are classified according to their length and include microRNAs (miRNAs) and long non-coding RNAs (lncRNAs) (reviewed in [137]).

### 4.1. MicroRNAs

MicroRNAs (miRNAs) are small (~22 nucleotides), ncRNAs that regulate their target mRNAs at the post-transcriptional level. miRNAs bind to the 3′-untranslated regions (UTRs) of target mRNAs, mediating translational repression or mRNA destruction [138,139]. A single miRNA can affect the expression of hundreds of targets [140], and multiple miRNAs can affect the same target. miRNAs play a key role in the development of human cancer with tumor suppressor miRNAs and oncogenic miRNAs (onco-miRs). To date, no HPV-encoded miRNAs have been discovered [141]. However, host miRNA expression is altered in the presence of HPV in cervical cancer tissue and precursor lesions, as well as in cervical cancer cell lines and keratinocytes expressing the HPV oncoproteins [142,143,144,145,146,147,148]. Moreover, a number of microRNAs, including miR-9, miR-21, miR-143, miR-203, and miR-372, among others, have been implicated in different aspects of cervical carcinogenesis, with the expression of some microRNAs increased (miR-21, miR-143, miR-9) and others decreased (miR-34a, miR-203, miR-372) [55,149,150,151,152,153,154]. Bioinformatic analyses of microRNA expression, coupled with changes in RNA expression as a result of HPV16E6/E7 in human keratinocytes, identified a number of canonical pathways targeted by miR-modulated mRNAs, including cyclins, cell cycle regulation, estrogen-mediated S-phase entry, and aryl hydrocarbon reception signaling [155]. Experiments to dissect the molecular mechanisms underlying the mode of action of particular microRNAs in cervical carcinogenesis revealed that miR-21 targets chemokine (C-C) motif ligand 20 (*CCL20*), and its overexpression regulates proliferation, apoptosis, and migration of HPV16-positive cervical cancer cells [156]. Increased levels of miR-203 inhibit HPV amplification, and HPV E7 suppresses miR-203 to allow for productive replication to occur [157]. mir372 is downregulated and targets CDK2 and Cyclin A1 in cervical cancer [152]. When comparing studies such as these, which focus on a single microRNA and the modulation of a single target mRNA, with studies that investigate the modulation of cellular microRNAs by HPV gene expression, one must take into consideration the global landscape of microRNA expression, the cell type studied (differentiating versus undifferentiated epithelial cells), the HPV type studied, and the whole HPV genome versus just HPV E6 and/or E7. In fact, these considerations should be kept in mind when comparing all of the studies mentioned in this review.

A number of miRNAs are epigenetically regulated, suggesting that aberrant methylation of miRNA promoters is one of the possible mechanisms for deregulation of miRNAs in cervical cancer [149,158,159]. The miRNA biogenesis machinery is often dysregulated in human cancers, including cervical carcinoma (reviewed in [160,161]). Chromosome 5p amplifications are found in some cervical carcinomas, and *DROSHA* is the most significantly overexpressed transcript in cervical tumors with 5p gain [162,163]. Expression of high-risk HPV E6 and E7 in HPV-negative C33A cervical carcinoma cells and primary human epithelial cells causes increased expression of *DROSHA* and *DICER* [164], and many *DROSHA*-regulated miRNAs are dysregulated in high-risk HPV16 E6/E7 expressing cells [155,164].

### 4.2. Long Non-Coding RNAs

Long non-coding RNAs (lncRNAs) are non-coding RNA transcripts with a length greater than 200 nucleotides; to date, 27,919 lncRNA have been discovered in humans [165]. Although their function is not fully elucidated, they do contribute to many biological processes including cellular development, differentiation, and transformation. However, it is known that lncRNAs bind to PRC1 and PRC2, function as antisense molecules, and organize enhancer activity (reviewed in [166,167]). A number of lncRNAs are differentially expressed in cancer, including HOX transcript antisense intergenic RNA (HOTAIR) [168,169,170,171,172,173]. HOTAIR regulates gene expression through association with chromatin remodeling complexes [174]; it bridges PRC2 with the lysine-specific histone demethylase1A complex (LSD1), resulting in gene silencing [168,174,175]. Down-regulation of HOTAIR, with corresponding upregulation of the HOTAIR target HOXD10, has been observed in cervical cancer [176].

## 5. Concluding Remarks

High-risk HPVs are associated with approximately five percent of human cancers, including virtually all cervical cancers, as well as anal, vaginal, vulvar, penile, and oropharyngeal cancers. Although highly efficacious prophylactic vaccines appear promising for preventing a large fraction of HPV-associated cancers, they do not protect from pre-existing infections or prevent malignant progression, and are not expected to impact the frequency of these cancers for decades. In the meantime, millions will develop HPV-associated cancers, and many will die of these cancers worldwide. It is imperative that we identify novel therapeutic targets to control and, ideally, eradicate HPV-associated cancers. A number of epigenetic alterations have been identified that occur in both the HPV and the cellular genome, including DNA hypomethylation, hypermethylation of tumor suppressor genes, histone modifications, and alterations in ncRNAs. These alterations have the potential to be used as biomarkers for early detection. In addition, epigenetic alterations, unlike genetic mutations, may be reversed by inhibiting the associated enzymes, and as such should be evaluated as therapeutic modalities for HPV-associated lesions and cancers. Moreover, we can apply the findings of these studies to other, non-HPV associated cancers.
